# Monensin reduces enteric methane emissions in late-lactation Holstein cows fed high-concentrate diets

**DOI:** 10.3168/jdsc.2025-0865

**Published:** 2025-10-30

**Authors:** D. Onan-Martinez, M.A.T. de Bari, H. Olmo, J. Lance, I.M. Toledo, J.M. Tricarico, G.E. Dahl

**Affiliations:** 1 Department of Animal Sciences, University of Florida, Gainesville, FL 32611; 2 Dairy Management Inc., Rosemont, IL 60018

## Abstract

Methane is a major GHG produced by cattle. Strategies to reduce enteric methane include supplementation with monensin, which has shown variable outcomes. Therefore, we hypothesized that monensin (Rumensin, Elanco, Greenfield, IN) would reduce enteric CH_4_ yield and intensity in lactating dairy cows (∼207 DIM). Twenty cows were enrolled in a 2 4-wk period crossover completely randomized design with repeated measures including 2 treatments: control (CON, n = 10/period) and monensin (MON, n = 10/period). Monensin treatment consisted of 300 mg of monensin in 34 g/cow of dried distillers grains (DDG) whereas CON was DDG alone, both top-dressed daily on the TMR. Cows were milked 3 times/d and housed in a sand-bedded freestall barn with Calan gates to collect individual DMI. A GreenFeed system was used to measure enteric gas output. Daily milk yield and DMI were measured from each cow during the study. Additionally, fat, protein and lactose percentages were measured weekly in milk. Average (±SEM) DMI (MON = 25.4 ± 0.3, CON = 25.4 ± 0.3, kg/d) and ECM (MON = 35.8 ± 1.09, CON = 36.4 ± 1.09; kg/d) were similar between treatments. Conversely, daily CH_4_ was significantly reduced with monensin (MON = 207.1 ± 13.1, CON = 257.2 ± 13.1 g/d). Methane yield showed a significant decline with treatment (MON = 8.1 ± 0.4, CON = 9.9 ± 0.4 g/kg DMI). Last, methane intensity relative to ECM tended to decline with monensin (MON = 5.7 ± 0.3, CON = 6.5 ± 0.3 g/kg of ECM). The results suggest that monensin reduces daily enteric CH_4_ emissions and yield in dairy cattle, providing evidence to support its use to reduce CH_4_ emissions from cattle.

## Highlights

• Monensin did not alter DMI in late-lactation dairy cattle.
• Milk production and milk components remained unaffected between treatments.
• Monensin decreased methane emissions and yield and tended to reduce methane intensity.
• The GreenFeed system is a reliable tool to measure enteric gases from cattle.
• GreenFeed measurement quality depends on cows' voluntary visits to the unit.

## Graphical Abstract

**Figure:**
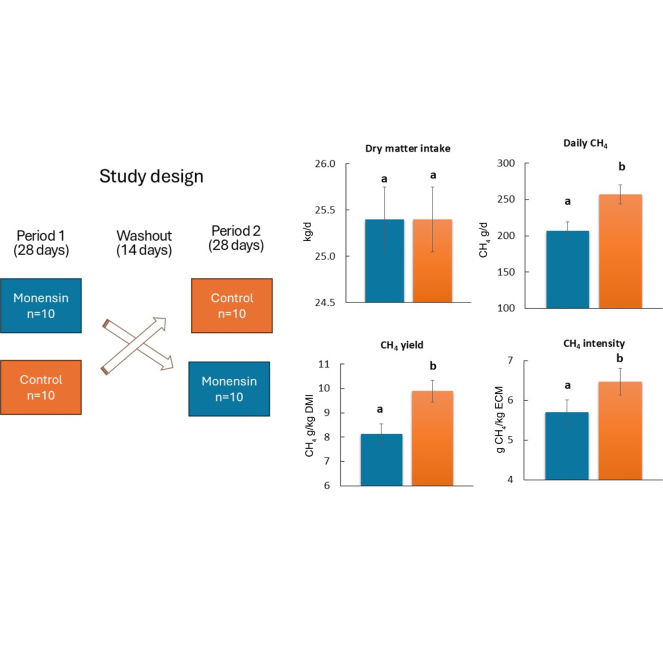
**Summary:** Methane is a major greenhouse gas that contributes to global warming. Cattle produce methane through enteric fermentation, and several strategies have been tested to reduce these emissions. Dietary supplementation with monensin has shown variable results. Therefore, our study evaluated its effects in late-lactation dairy cattle. We measured individual feed intake, milk yield and components, and methane emissions using a GreenFeed system in a 2-period crossover completely randomized design involving 20 cows (monensin = 10, control = 10). We found that monensin did not alter dry matter intake (DMI), milk production, or milk components. However, daily methane emissions, methane yield, and methane intensity were reduced. Our findings support the use of monensin to mitigate methane emissions from dairy cattle without affecting productivity. Error bars represent standard error of the mean. Least squares means values represented in bars with different letters (a,b) differ (*P* < 0.10). ECM = energy-corrected milk.

Methane is a waste product from microbial fermentation of organic matter in the rumen (Khan et al., 1997). Methanogenic archaea are microorganisms found in the rumen that can generate CH_4_ by converting CO_2_ and H_2_. Their role is crucial for keeping hydrogen levels low in the rumen, which supports the growth of other species and ensures efficient fermentation of feed (Matthews et al., 2019). However, CH_4_ is also considered a loss of energy estimated between 2% to 12% of gross intake in cows (Johnson and Johnson, 1995). Most of CH_4_ in the rumen is generated by the hydrogenotrophic pathway; however, a fraction is generated from the methylotrophic and acetoclastic pathways (Hill et al., 2016).

Reducing methane emissions from cattle could benefit the environment (Wallace et al., 2014). Although the search for strategies to reduce enteric CH_4_ is active, it remains highly challenging. Researchers have explored various approaches, such as selective breeding, vaccines, methanogenesis inhibitors, and dietary interventions, with most efforts focusing on directly inhibiting methanogenesis through dietary compounds (Glasson et al., 2022). Dietary compounds must meet several criteria to ensure their effectiveness and safety for animals, humans, and the environment. Furthermore, they must undergo rigorous regulatory testing before becoming commercially available. Although evaluation and classification criteria vary across countries and regions, there is a consistent global reliance on robust scientific evidence before adoption (Tricarico et al., 2025).

Ionophores are commercially available to improve feed efficiency and for prevention and control of coccidiosis. However, its potential for methane reduction has been tested with variable results (Sauer et al., 1998; Odongo et al., 2007; Benchaar, 2016). Ruminal protozoa and gram^+^ bacteria are particularly sensitive to monensin, resulting in disruption of protozoa-methanogen archaea interaction and lowering CH_4_ production. Additionally, sensitivity of acetate-producing bacteria (gram^+^) reduces hydrogen availability for CH_4_ synthesis. (Russell and Strobel, 1989).

Researchers have examined the effect of monensin on feed intake and milk yield, with some studies indicating no effect (Martineau et al., 2007; Vendramini et al., 2016) and others reporting positive outcomes on feed efficiency and milk yield (Phipps et al., 2000). The variability in monensin effects on lactating cows appears to be linked to the stage of lactation and dose (Rezaei Ahvanooei et al., 2023). Early- and mid-lactation cows benefit the most because their energy balance is negative or barely positive because of increasing and peaking milk production (Rezaei Ahvanooei et al., 2023). Moreover, supplementation of less than 16 mg/kg shows no effect, whereas doses between 16 and 21 mg/kg improve lactation performance, especially in early to mid-lactation. However, doses above 37 mg/kg result in detrimental effects on DMI and milk yield (Rezaei Ahvanooei et al., 2023). The effects of monensin on CH_4_ emissions are also variable potentially due to diet composition, level of intake, and dose differences across studies. For instance, Sauer et al. (1998) reported a 21% reduction in daily CH_4_ emissions whereas Odongo et al. (2007) found 7% reduction in daily CH_4_ emission, but Benchaar **(**2016) did not observe differences in daily CH_4_ nor yield emissions. Finally, studies have been conducted in late-lactation cows, but no CH_4_ measurements were collected (Bell et al., 2006; Mathew et al., 2011). Because of the variable results observed across the literature on the effects of monensin on methane emissions and few studies testing monensin on late-lactation cows we hypothesized that monensin (Rumensin, Elanco, Greenfield, IN) reduces enteric methane emissions from late-lactation dairy cattle.

This study was conducted at the University of Florida Dairy Unit (Alachua, FL) with the approval of all procedures by the Institutional Animal Care and Use Committee. A total of 20 lactating Holstein cows with an average of 207 DIM (SD = 24) were enrolled in a completely randomized study with a 2-period crossover design. In each 4-week period, 10 random cows (**MON**) were supplemented with 300 mg/cow of monensin sodium in 34 g/cow of dried distillers grains (**DDG**) and a second group of 10 random cows (**CON**) only received DDG. Treatments were applied nonblinded and simple randomization using Microsoft Excel (version 365, 2023, Microsoft Corporation, Redmond, WA) was performed to assign cows to treatments. Both treatments were top-dressed daily on the TMR. A 14-d washout period, during which no treatments were given to any of the cows to eliminate potential carryover effects, was implemented between the two 4-wk treatment periods. Cows were housed in freestall barns equipped with automatic activated gates for each bunk (Calan Gate System) to measure individual feed intake. Cows were fed a ration formulated to meet their requirements. However, due to limited forage availability at the time of the experiment, we fed a diet with concentrate inclusion of 60.3% and 39.7% forage on a DM basis which is above the typical composition of the regular diet at our farm (usually 45% grain mix and 55% forage; Table 1). Cows were fed ad libitum, and their individual feed intake was calculated by offering feed in the morning and afternoon and measuring feed refusals after 24 h relative to the morning feeding.

**Table 1:** Diet ingredients fed to the cows supplemented with and without monensin throughout two 28-d periods, including calculated nutrient analysis on a DM basis^1^

Item	Inclusion, % of DM
Ingredient	
Corn meal	22.7
Soybean	11.6
Aminoplus^2^	5.7
Nurisol^3^	0.4
Wheat straw	4.0
Corn silage	35.7
Citrus pulp	11.9
Palmit 80^4^	2.1
Lactating cow mineral mix^5^	5.9
Calculated nutrient analysis, % of DM	
Forage inclusion	39.7
CP	16.06
MP allowable milk, kg/d	49.2
ME allowable milk, kg/d	44.9
Sugar	5.63
aNDFom^6^	24.63
Ether extract	5.12
Starch	29.73

1 The MON group (n = 20) was supplemented with 300 mg of monensin in 34 g/cow of DDG and the CON group (n = 20) received 34 g/cow of DDG alone, both top-dressed on TMR.

2 Aminoplus (bypass soybean meal), Ag Processing Inc., Omaha, NE.

3 Nurisol (calcium salt of long-chain fatty acids), Global Agri-Trade, Rancho, CA.

4 Palmit 80 (palm fatty acid distillate), Global Agri-Trade, Rancho, CA.

5 Lactating cow mineral mix contained 9.8% Ca, 1.9% P, 2.5% Mg, 0.1% S, 0.7% K, 7.2% Na, 1,151.6 mg/kg Zn, 182.6 mg/kg Cu, 472.2 mg/kg Fe, 933.3 mg/kg Mn, 18.1 mg/kg Co, 10.4 mg/kg I, 6.1 mg/kg Se, 100,823 IU of vitamin A/kg, 53,257 IU of vitamin D/kg, and 455 IU of vitamin E/kg (DM basis).

6 aNDFom = amylase-treated NDF, OM basis.

Cows were milked 3 times per day, and milk yield was recorded throughout the study using an AfiLab milk analyzer (Afimilk Agricultural Cooperative Ltd., Kibbutz, Israel). In addition, milk samples were collected once a week from each cow using 50-mL tubes containing bronopol B-14 pills as a preservative. Milk component analysis included percentages of fat, protein, and lactose at the Southeast Milk Inc. Laboratory (Belleview, FL). Subsequently, ECM was estimated using the following formula: ECM, kg/d = [(0.3246 × milk yield) + (12.86 × fat yield) + (7.04 × protein yield)] per NRC (2001).

To measure enteric gas output from individual animals, we used a GreenFeed System Unit (**GF**; C-Lock Inc., Rapid City, SD). GreenFeed units are automated head chambers that collect short-term measurements of gases from individual cows allowing for on-site measurements without altering the housing or normal management. Animals are attracted to the unit by a small amount of bait feed released from a hopper. In the present study, we used soyhull pellets as bait to attract the cows to the GF unit. The unit was set to allow the cows up to 9 bait drops per visit, and 6 visits per day with a 3-h interval between visits. The objective of this setting was to obtain high quality visits of around 3 min or more to be considered a representative sample, dispersed throughout the day. The gases measured from the animals were CH_4_, CO_2_, and hydrogen H_2_.

The experiment was a crossover arrangement in a completely randomized design with repeated measures where the individual cow was the experimental unit. Data were analyzed using the PROC MIXED procedure of SAS (version 9.4, SAS Institute Inc., Cary, NC). The model considered fixed effects of treatment, day, period, sequence, and treatment by day interaction and the random effect of cow within sequence. Predicted transmitting ability was included in the initial model, but it was not significant for any of the variables, including intake, ECM, and milk components, and therefore it was removed. Data are presented as LSM with SEM. Significance was considered when *P* < 0.05 and tendency when *P* ≤ 0.10, and residuals were tested for normality.

Dry matter intake did not differ between MON and CON (MON = 25.4 ± 0.3 kg/d, CON = 25.4 ± 0.3 kg/d; *P* = 0.85). Additionally, milk yield (MON = 34.7 ± 1.47 kg/d, CON = 35.0 ± 1.47 kg/d; *P* = 0.46) and ECM (MON = 36.4 ± 1.09 kg/d, CON = 35.8 ± 1.09, kg/d; *P* = 0.34) were not different between treatments. Similarly, milk components were not different between treatments (Table 2).

**Table 2:** Dry matter intake, milk yield, milk components, daily methane, methane yield and intensity, carbon dioxide, and hydrogen emissions from cows with and without monensin supplementation during a crossover arrangement (two 28-d periods) study^1^

Response	Treatment		SEM	*P*-value
	CON	MON		
DMI, kg/d	25.4	25.4	0.3	0.85
Milk yield, kg/d	34.7	35.0	1.47	0.46
ECM, kg/d	36.4	35.8	1.09	0.34
Efficiency, ECM/DMI	1.38	1.39	0.06	0.41
Milk fat, %	3.89	3.92	0.17	0.83
Milk protein, %	3.21	3.21	0.08	0.95
Milk lactose, %	4.69	4.72	0.04	0.30
Visits to GF unit,^2^ visits/cow per d	1.4	1.3	0.2	0.33
Methane,^2^ g/d	257.2^a^	207.1^b^	13.1	0.002
Methane yield,^2^ g/kg of DMI	9.9^a^	8.1^b^	0.4	0.004
Methane intensity,^2^ g/kg of ECM	6.5^a^	5.7^b^	0.3	0.08
Carbon dioxide,^2^ g/d	12,125	11,761	332	0.26
Hydrogen,^2^ g/d	1.73	1.40	0.18	0.13

1 The MON group (n = 20) was supplemented with 300 mg of monensin in 34 g/cow of DDG and the CON group (n = 20) received 34 g/cow of DDG alone, both top-dressed on TMR. Enteric gases were measured with a GreenFeed system. *P*-values for treatment effects are included.

2 Gas measurements of 4 cows were included in the analysis as missing values because they refused to visit the unit during the trial (3 cows for the CON-MON sequence and 1 for the MON-CON sequence).

All cows were trained to the GF unit for 15 d before the trial started, yet there were 4 cows that refused to visit the unit during the trial (3 cows for the CON-MON sequence and 1 for the MON-CON). Because no gas measurements were obtained from them, but all other variables were still collected, gas measurements from these cows were included as missing values. We obtained a total of 646 valid visits during the length of the experiments with an average 03:44-min duration per visit. The diurnal pattern of visits and CH_4_ emission average during the length of the experiment is presented per hour of the day in Figure 1. There was no significant difference in visits between groups (MON = 1.3 ± 0.2, CON = 1.4 ± 0.2, valid visits/d; *P* = 0.33), yet a significant period effect was observed negatively affecting visits (period 1 = 1.8 ± 0.2, period 2 = 0.9 ± 0.2, valid visits/cow per d; *P* < 0.01). The importance of visits will be discussed later in this paper.

**Figure 1:**
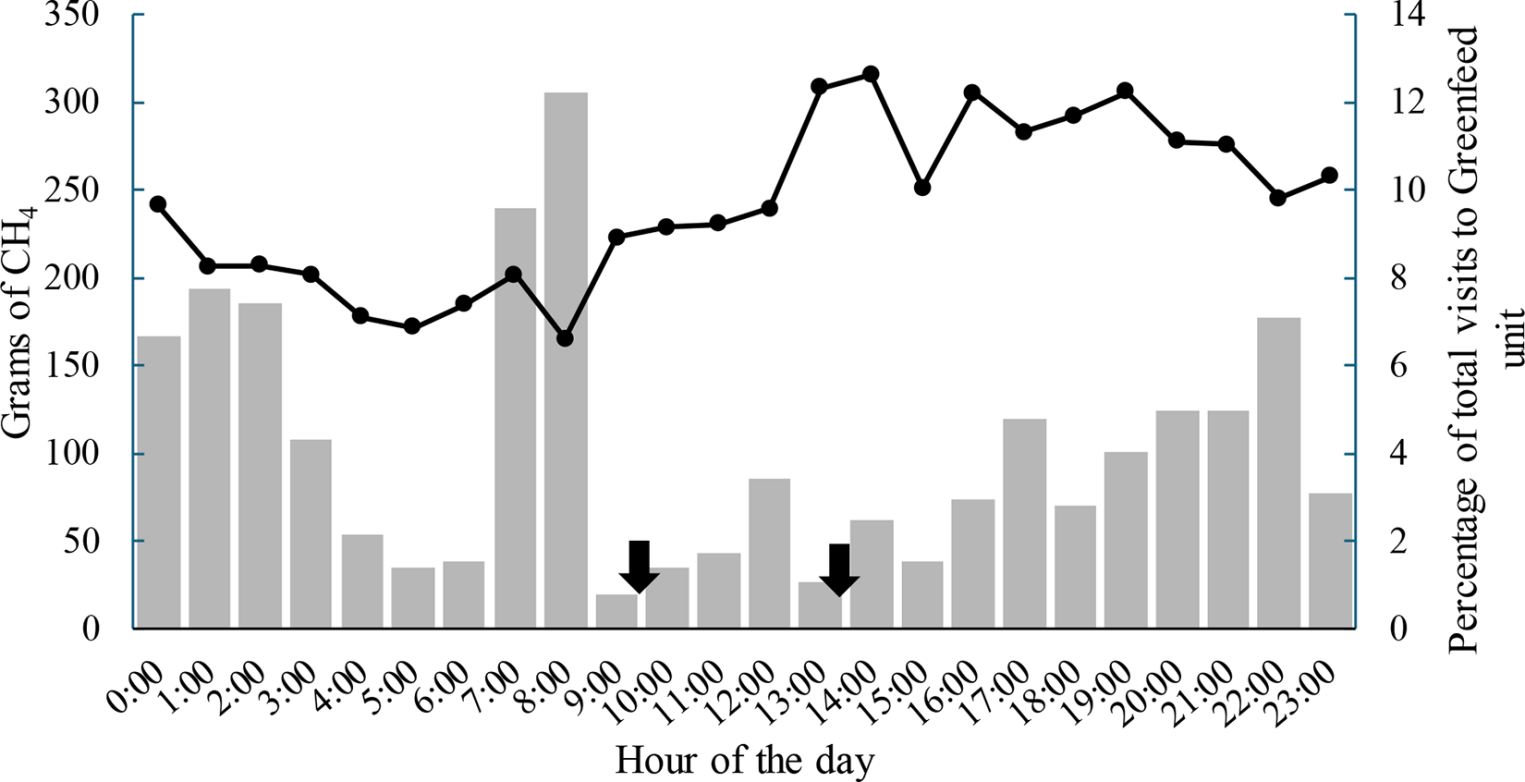
Diurnal pattern of visits to the GreenFeed unit (bar graph) and methane emissions (continuous line with solid circles) from cows in both groups, the MON group (n = 20), which was supplemented with 300 mg of monensin in 34 g/cow of DDG, and the CON group (n = 20), which received 34 g/cow of DDG alone, during the two 28-d periods of the study. Visits are reported as percentage per hour of total visits during the experimental period. Arrows indicate feeding time.

Gas measurements are summarized in Table 2. Monensin supplementation in the diet decreased daily CH_4_ emissions (*P* = 0.002) compared with CON cows (MON = 207.1 ± 13.1 g/d, CON = 257.2 ± 13.1 g/d). Similarly, methane yield (i.e., grams of CH_4_/kg of DMI) was significantly reduced (*P* = 0.004) by MON relative to CON (MON = 8.1 ± 0.4 g/kg DMI, CON = 9.9 ± 0.4 g/kg DMI), whereas methane intensity (i.e., grams of CH_4_/kg of ECM) tended to decrease (*P* = 0.08) with MON supplementation (MON = 5.7 ± 0.3 g/kg of ECM, CON = 6.5 ± 0.3 g/kg of ECM). However, CO_2_ and H_2_ emissions were not different between groups (*P* > 0.1).

Monensin supplementation did not alter DMI, milk yield, ECM, or feed efficiency compared with control treatment in the present study. Our results align with those of Martineau et al. (2007) and Vendramini et al. (2016), who found no difference in DMI or milk yield with monensin supplementation. However, Phipps et al. (2000) and more recently Rezaei Ahvanooei et al. (2023) reported that monensin supplementation decreased DMI and increased milk yield relative to control groups. Variability of monensin's effects in lactating cows seems to be related to lactation stage, where early- and mid-lactation cows benefit the most, likely because energy balance is negative or barely positive as milk production is increasing and reaching a peak, whereas in late lactation, milk production is declining. Therefore, as observed in our study, it is expected that in late lactation milk yield will not be affected by monensin and, consequently, intake and efficiency are not affected either. However, our sample size might have limited the ability to detect differences in intake. Moreover, the effects of monensin are dose-dependent because supplementation of less than 16 mg/kg is without effect in production parameters as in our case where dosage falls in this category (i.e., 11.8 mg/kg), whereas doses of 16 to 21 mg/kg improve lactation performance, especially from 60 to 150 DIM. However, doses exceeding 37 mg/kg result in detrimental effects on lactation performance (Phipps et al., 2000; Rezaei Ahvanooei et al., 2023).

As with intake and milk yield, no differences in milk components were observed between groups. Variable results have been reported across experiments testing the effects of monensin on milk components, with milk fat being the most variable. Odongo et al. (2007) and Rezaei Ahvanooei et al., 2023 reported fat and protein depression due to monensin supplementation. However, Vendramini et al. (2016) reported no differences in milk components. The dose of monensin we used might have been too low to induce changes in milk fat. Additionally, like Vendramini et al. (2016), our study used late-lactation cows, which might have contributed to the lack of effect on milk fat.

GreenFeed is a system that bases CH_4_ measurements on voluntary animal visits to the instrument, which can represent a limitation when cow visits are not frequent enough. In the present study, visit number did not differ between groups (MON = 1.3 ± 0.2, CON = 1.4 ± 0.2, valid visits/d; *P* = 0.33). However, each cow was allowed to visit up to 6 times per day. Moreover, a significant period effect was observed, which negatively affected visits during period 2 (period 1 = 1.8 ± 0.2, period 2 = 0.9 ± 0.2, valid visits/cow per d; *P* < 0.01). This unexpected drop might be explained by increasing temperatures as we were transitioning from spring (May, period 1) into summer (June, period 2) of 2023. Summer heat load is a major problem for dairy cows in Florida where, despite the implementation of evaporative cooling systems, cows still undergo significant levels of heat stress that induce negative behavioral (Toledo et al., 2023) and production (Ferreira and De Vries, 2015) shifts that might explain fewer visits during period 2 in both groups. Despite the fewer number of visits in period 2, a total of 646 valid visits were obtained during the entire experiment, which is comparable to other studies where fewer animals were enrolled over a longer period and visit number (average and total visits) was similar (Coppa et al., 2021; Kamalanathan et al., 2023). In contrast, other studies showed a larger number of visits (Alemu et al., 2017; Hristov and Melgar, 2020), which might represent improved estimates of gas output. By analyzing the different scenarios relative to the total number of visits in other studies as well as manufacturer recommendations, we consider that differences in breeds (beef or dairy), housing systems, number of animals per GF unit, location and time availability of GF unit, and type of bait across experiments likely contributed to the variability in visit number (Alemu et al., 2017; Hristov and Melgar 2020; Coppa et al., 2021; Kamalanathan et al., 2023). Based on our results, we speculate that heat stress might influence visit frequency but this has not been tested. Across multiple studies, GF visit number is one of the most important factors in accurate emission estimates, so aiming for a greater number of sampling points is a priority. Starsmore et al. (2024), stated an adequate visit frequency as >1 visit/d and obtained 2.2 visits/cow per d, whereas Vargas et al. (2024) recommend a total of 60 visits per cow throughout an experiment. Although C-Lock (Rapid City, SD) does not state a specific visit frequency requirement, they do recommend multiple visits throughout the day for at least 1 wk to ensure representative data. Visit behavior varied across the day in our study, where cows visited more frequently from 2400 to 0300 h, then from 0700 to 0800 h and finally again at 2200 h (Figure 1). An increase in visits overnight was also observed by Alemu et al. (2017), but they did not speculate on an explanation for the pattern. Given the environmental conditions in our study, especially the low number of visits in period 2 that matched with increasing temperatures, the diurnal distribution of visits may be associated with heat stress effects on DMI. Alternatively, the 0700 to 0800 h increase occurred right before the first feeding of the day, and a nadir of visits was observed after both feeding times, which might mean that cows opted to get bait from the GF unit, especially before the initial TMR feeding. This could be a concern considering that before the first feeding ruminal fermentation rate would be expected to be low, and if most visits occur at this time, the daily average of emissions might be skewed. Indeed, this might be one of the factors driving the lower daily methane emissions when compared with other studies as discussed later in this paper. Consistent with a feeding time effect, an increase in CH_4_ emissions was observed immediately after both feeding times, indicating that fermentation rate in the rumen increases as substrate is provided, resulting in a diurnal emissions pattern that compares with other studies using the gold standard for CH_4_ emissions measurements, respiration chambers (Judy et al., 2018; Benchaar and Hassanat, 2020).

Daily methane emissions, and methane yield were decreased by 19.5% and 18.2%, respectively, while intensity tended to decrease by 12.3% with monensin supplementation relative to control treatment. Our findings align with those of Sauer et al. (1998), Odongo et al. (2007), and Ranga Niroshan Appuhamy et al. (2013), who reported reductions that range from 7% to 21% for daily methane. In contrast, Benchaar (2016), did not observe reductions of methane emissions. Methane reduction might be explained by the effect of monensin and high-grain diets on the VFA profile induced by favoring propionate-producing bacteria. Propionate production is a sink for hydrogen ions, which results in less hydrogen available for its reduction into methane, and therefore the increased propionate production indirectly reduces methane production (Russell and Strobel, 1989). As discussed previously, this effect on methane output may vary due to diet composition, level of intake, and supplementation dose differences across studies (Beauchemin et al., 2009; Rezaei Ahvanooei et al., 2023). Despite the comparable reduction in average daily methane due to MON, the average emissions in both MON and CON cows seemed to be lower when compared with other studies where cows had similar levels of DMI (Odongo et al., 2007: Hristov and Melgar 2020; Coppa et al., 2021). As explained previously, cows visited the unit less than expected, especially in period 2. Additionally, there was a higher number of visits at night and right before first feeding. Moreover, our cows were fed a high-grain diet that might have contributed to shifts toward propionate contributing to further lower emissions. All together, these factors might have decreased the daily CH_4_ emissions captured by GF from our cows and consequently, CH_4_ yield and intensity.

Carbon dioxide emissions did not differ between groups in our study. Variability of CO_2_ was lower than that of CH_4_, and CO_2_ production was proportional to energy expenditure (i.e., milk production and activity; Alemu et al., 2017), therefore we expected that because in the present study both groups had similar intake, production levels, and the same housing conditions, CO_2_ would be similar as well. The present findings were consistent with those reported by Sauer et al. (1998) and Vyas et al. (2018).

In the present study, H_2_ was not different between groups, a result that differs from the results of the Vyas et al. (2018) study, where hydrogen emissions were reduced by monensin supplementation. Monensin increases the propionate production pathway in which hydrogen is sequestered, thereby reducing hydrogen emissions. However, the lack of difference in H_2_ in our study might be explained by the alternate H_2_ sink pathways such as formate, valerate, caproate, heptanoate, UFA, nitrate and sulfate reduction, and microbial protein synthesis (Vyas et al., 2018).

Monensin is a widely used ionophore that has shown improvements in efficiency of dairy and beef cattle (Rezaei Ahvanooei et al., 2023). However, in the present study differences in intake, milk production, and milk components were not evident, potentially due to the stage of lactation of the cows, variations in diet, and dose of supplementation when compared with other studies. Yet methane emissions expressed as daily CH_4_, yield and intensity were clearly reduced by monensin, demonstrating its potential as a methane-reduction strategy.
